# Persistent perceptual delay for head movement onset relative to auditory stimuli of different durations and rise times

**DOI:** 10.1007/s00221-012-3112-x

**Published:** 2012-05-13

**Authors:** Michael Barnett-Cowan, Sophie M. Raeder, Heinrich H. Bülthoff

**Affiliations:** 1 Department of Human Perception, Cognition and Action, Max Planck Institute for Biological Cybernetics, Tübingen, Germany; 2 Department of Brain and Cognitive Engineering, Korea University, Seoul, South Korea; 3 Present Address: Department of Psychology, The Brain and Mind Institute, Western University, London, ON Canada

**Keywords:** Auditory, Duration, Head movement, Multisensory, Subjective simultaneity, Temporal order judgment, Time perception, Vestibular

## Abstract

The perception of simultaneity between auditory and vestibular information is crucially important for maintaining a coherent representation of the acoustic environment whenever the head moves. It has been recently reported, however, that despite having similar transduction latencies, vestibular stimuli are perceived significantly later than auditory stimuli when simultaneously generated. This suggests that perceptual latency of a head movement is longer than a co-occurring sound. However, these studies paired a vestibular stimulation of long duration (~1 s) and of a continuously changing temporal envelope with a brief (10–50 ms) sound pulse. In the present study, the stimuli were matched for temporal envelope duration and shape. Participants judged the temporal order of the two stimuli, the onset of an active head movement and the onset of brief (50 ms) or long (1,400 ms) sounds with a square- or raised-cosine-shaped envelope. Consistent with previous reports, head movement onset had to precede the onset of a brief sound by about 73 ms in order for the stimuli to be perceived as simultaneous. Head movements paired with long square sounds (~100 ms) were not significantly different than brief sounds. Surprisingly, head movements paired with long raised-cosine sound (~115 ms) had to be presented even earlier than brief stimuli. This additional lead time could not be accounted for by differences in the comparison stimulus characteristics (temporal envelope duration and shape). Rather, differences between sound conditions were found to be attributable to variability in the time for head movement to reach peak velocity: the head moved faster when paired with a brief sound. The persistent lead time required for vestibular stimulation provides further evidence that the perceptual latency of vestibular stimulation is greater than the other senses.

## Introduction

Multisensory integration allows for a more coherent perception of our surroundings (Ernst and Bülthoff 2004). The ability to discern the temporal order of different stimuli is an important aspect of integration. Temporal asynchronies between the different sensory modalities result from differences in the propagation of different energies, as well as stimulus parameters. This poses a challenge for the brain to maintain a perception of simultaneity (see Vroomen and Keetles 2010 for a review). A turn of the head evokes a flood of time-varying sensory signals, which the brain must account for in order to maintain perceptual stability. The causal nature of this relationship suggests that the time at which a movement occurs is crucially important. However, large distortions of perceived auditory space during rapid head turns have been reported in the literature (Cooper et al 2008). Further, simultaneous occurrence of stimuli does not necessarily induce a corresponding perception of simultaneity (Spence and Squire 2003). Each sensory signal holds particular temporal properties, and temporal differences in sensory processing may be attributed to the physical properties of the different sensory systems, specifically transduction latencies (Pöppel et al. 1990) and axonal length (von Bekesy 1963). Cognitive factors, such as attention, have likewise been shown to affect processing time, with attended stimuli resulting in faster processing times (Spence et al. 2001). Specific stimulus parameters must also be considered such as stimulus intensity, which shows an inverse relationship with processing time (Efron 1963; but see Woodworth and Schlosberg 1954), stimulus envelope shape, where stimuli with rising onsets and falling offsets can be perceived as occurring either before or after a comparison square-shaped envelope stimulus (Vos and Rasch 1981; Jaśkowski 1993), and stimulus duration, where a shorter stimulus can shift toward the offset of its paired longer stimulus (Jaśkowski 1991, 1992; but see Efron 1970a, b, c as well as Boenke et al. 2009 who found no such effects of duration).

It has recently been shown that the perceived onset of vestibular stimulation is slow with respect to the other senses (Barnett-Cowan and Harris 2009, 2011; Barnett-Cowan et al. 2010; Sanders et al. 2011). Vestibular perception appears to be delayed anywhere from 50 to 200 ms in relation to auditory perception (Barnett-Cowan and Harris 2009, 2011; Sanders et al. 2011), as reflected by the point of subjective simultaneity (PSS). The PSS is defined as the perceptual temporal asynchrony between two stimuli occurring simultaneously. This delay is surprising considering the low latencies involved in the transduction (~40 μs; Corey and Hudspeth 1979) and physiological response (~20 ms latency of the vestibular ocular response; Lorente de No 1933) to vestibular stimulation. One possible explanation for this finding is that these previous studies compared vestibular stimulation of long duration (~1 s) and of a continuously changing temporal envelope with a comparison stimulus that was briefly (10–50 ms) pulsed. While there is no reason to suspect that the duration or shape of the temporal envelope of a comparison stimulus would interact with vestibular perception when determining onset simultaneity, particularly as such effects have been observed for unimodal stimulus pairs, it is important to assess this hypothesis given the unexpected delay that has been observed for the perceived timing of vestibular stimulation.

In order to determine the potential effect of these methodological constraints, we first had participants make non-speeded temporal order judgments (TOJs) comparing the perceived onset of active head movement with the onset of brief (50 ms) and long (1,400 ms) sound, with long sounds having either a square- or a raised-cosine-shaped envelope. Here, a raised-cosine-shaped envelope was selected as it reasonably approximates change in head position when rotating back and forth. The predictions that we assessed were that (1) head movement has to precede a brief square sound and (2) less or no head movement lead time would be required when paired with long square and/or long raised-cosine sounds where comparable durations would not lead to erroneous shifts of the PSS (Jaśkowski 1991, 1992) and where a longer rise time would be expected to delay the perceived onset of the auditory stimulus (Vos and Rasch 1981). Thus, if the PSS shifts toward or past a stimulus onset asynchrony (SOA) of 0—the point of true simultaneity—as the comparison stimulus characteristics resemble those of a head movement more, then temporal processing differences between vestibular and auditory stimulations would likely be attributable to differences in stimulus characteristics. Alternatively, failure of the PSS to shift toward an SOA of 0 would support previous work, indicating that vestibular processing is delayed relative to the other senses. In a second experiment, participants made TOJs among the auditory stimuli.

## General methods

### Participants

Thirteen German diploma students visiting the Max Planck Institute for Biological Cybernetics and two authors (SMR and MB-C) participated in the study (four women, aged 22–53 years) and gave their informed written consent in accordance with the ethical standards specified by the 1964 Declaration of Helsinki prior to their inclusion in the study. Participants reported having no auditory, vestibular or other neurological disorders.

### Head movement generation recording and analysis

Active head movement was self-generated by participants and monitored using an eye tracker equipped with inertial motion sensors (Chronos Vision GmbH, Berlin, Germany). Signals from the inertial motion sensors were fed to a PC (Windows 2000) via an analog-to-digital converter and recorded at 100 Hz using software supplied from the manufacturer (ETD version 3703). The eye tracker was secured to the head with a custom-made chin strap in addition to the supplied forehead strap (c.f. Barnett-Cowan and Harris 2008). Note that eye movements were not actually recorded. The onset of head movement was defined in post hoc analysis as having occurred when the head moved at a velocity greater than 2 SD from the average head velocity recorded in the first 100 ms of each trial while the head was stable (c.f. Barnett-Cowan and Harris 2011). Trials in which the head moved during the first 100 ms of the trial were eliminated.

### Sound generation

Auditory stimuli were generated using Matlab 2006a and presented through earphones via an audio card (M-Audio Delta 1010LT) of a second PC (Windows 2000). A synchronized copy of this signal generated using the same audio card was sent via an analog-to-digital converter to the first PC recording head movement so that the two signals were recorded synchronously. Brief sounds were sinusoid waveforms with a duration of 50 ms consisting of a square envelope (5 ms rise-and-fall time) presented at 2,000 Hz. Long square sounds were sinusoid waveforms with a duration of 1,400 ms consisting of a square-shaped envelope stimulus (5 ms rise-and-fall time) presented at 2,000 Hz. Long raised-cosine sounds were sinusoid waveforms consisting of a raised-cosine envelope (Eq. 1) stimulus presented at 2,000 Hz rising to peak amplitude (α) for 1,400 ms (where μ is equal to 700 ms). Peak amplitude for all sounds was set at 80 dB SPL as measured for the duration of the long square sound. The justification for using such a loud stimulus was that we wanted to ensure that the onset of all stimuli would rapidly rise above threshold

1 $$ y = \frac{a}{2}\left[ {1 + \cos \left( {\frac{x - \mu }{\mu }\pi } \right)} \right]. $$

## Experiment 1

### Procedure

Prior to experimental trials, participants sat in a chair facing a fixation point and were instructed to rotate their head to a second fixation point located 20° to the right and then back again to the left. A sound (1,000 Hz sinusoidal waveform; 80 db; 50 ms), repeated every 700 ms, was used as a reference for the speed with which to move the head, such that participants were instructed to face each fixation point on every beat. Thirty beats were presented in total during this acoustical training period prior to each block, and participants were instructed to move their heads in accordance with this trained speed and displacement for the subsequent experimental trials.

**Fig. 1:**
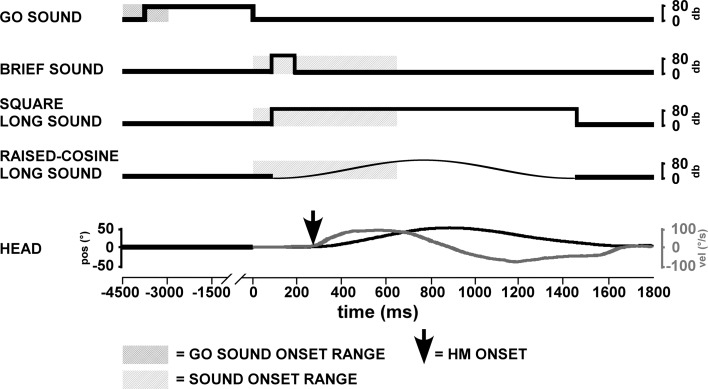
Trial design schematic. The trial begins with the offset of a go sound (200 Hz; time: 0 ms). The onset of either a brief square sound (50 ms, 2,000 Hz), a long square sound (1,400 ms, 2,000 Hz) or a long raised-cosine sound (1,400 ms, 2,000 Hz) occurred anywhere from 0 to 650 ms thereafter. The two traces in the *lower panel* show the position (*black line*, *left*-hand scale) and velocity (*gray line*, *right*-hand scale) of a typical head movement. The point of onset of head movement (indicated by the *arrow*) was defined in post hoc analysis (see “General methods”)

Figure 1 schematically shows the presentation of the stimuli in each trial. For each trial, participants were instructed to move their heads to the right and then back again to the left at the trained speed. Head movements were made following the offset of a “go” sound (200 Hz—*not* 2,000 Hz—sinusoidal waveform, 80 db), which also triggered a comparison stimulus. The duration of the go stimulus (i.e., intertrial interval) was 3 s, with an additional random 0–1.5 s duration to prevent anticipatory head movements. On account of the reaction time latencies relative to the go signal, comparison stimuli could occur before or after the head movement (c.f., Barnett-Cowan and Harris 2011). A comparison sound stimulus was presented between 0 and 650 ms after the go stimulus offset.

After each trial, participants were asked “which stimulus started first?” Participants responded by pressing either the left or right arrow key on a keyboard to indicate “sound first” or “head movement first”, respectively. Participants were instructed to attend equally to the sounds and head movement. There were three experimental blocks where, in each block, head movement was paired with one sound condition (one block containing brief sounds, one containing long square sounds, and one containing long raised-cosine sounds). The order of these three conditions was randomized across participants. There were 100 experimental trials in each block, which were preceded by 10 practice trials. Participants closed their eyes after being trained to move their head at a given speed and kept them closed for the duration of each block. Data collection took approximately 12 min for each block. Participants were allowed to take as long as they needed to make their judgments. The order of conditions was randomized across participants, and testing occurred within 1 h.

### Data analysis

The percentage of responses in which sound was selected as occurring first was plotted as a function of SOA, with negative SOAs signaling that the head movement took place before the presentation of the sound. A two-parameter sigmoidal logistic curve (Eq. 2) was fitted to the data using SigmaPlot (version 9). The inflection point of the logistic curve $(x_{0} )$ was taken as the point of subjective simultaneity (PSS), and the standard deviation (b) was taken as a measure of the just noticeable difference (JND), which provides an index of precision

2 $$ y = \frac{100}{{1 + e^{{ - \left( {\frac{{x - x_{0} }}{b}} \right)}} }}\%. $$

Statistical analysis included one-sample *t*-tests for the PSSs of each condition to confirm significant deviations from an SOA of 0 (i.e., the point of true simultaneity). A one-way repeated-measures ANOVA was carried out to examine differences in the PSSs and JNDs due to temporal envelope shape and duration of the auditory stimuli. Bonferroni’s adjustments were made for pairwise comparisons between means. For the data in which the normal distribution could not be assumed, a nonparametric Friedman’s test was employed.

### Results

#### Differences in PSS

**Fig. 2:**
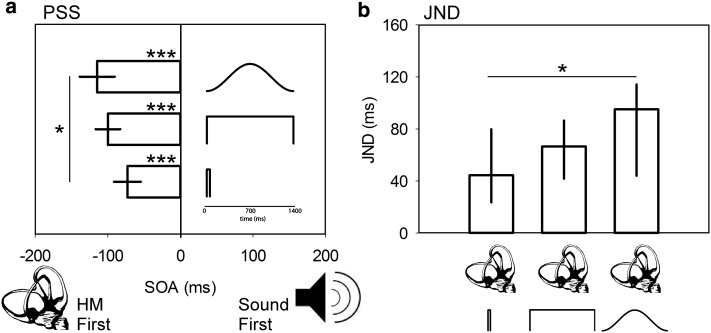
Experiment 1 results. **a** Average PSS plotted relative to SOA for each sound condition paired with a head movement (*HM*). Note that the cartoons representing each sound condition have a separate *inset* timescale than that used for SOA. *Error bars* are ±1 SEM. **b** Median JND data for each HM–sound pair. *Error bars* here are the 25th and 75th percentiles. *p < 0.05, ***p < 0.001

The average PSSs derived from TOJs for active head movement paired with brief (−73.0 ms, s.e. 18.4), long square (−99.8 ms, s.e. 16.9) and long raised-cosine (−114.7 ms, s.e. 24.2) sounds are shown in Fig. 2a. Pairwise comparison tests confirmed that the significant effect of sound type on the PSS (*F*_(2,28)_ = 3.5, *p* = 0.043) was driven by the long raised-cosine sound, which was significantly different from brief square sounds (*p* = 0.042) but not from the long square sounds (*p* = 0.358). The difference in PSS between brief and long square sounds was not significant (*p* = 0.196). All PSSs were significantly different from true simultaneity (one-sample *t*-tests, all *p* < 0.001), confirming that head movement must precede all sound types in order to be regarded as simultaneous.

#### Differences in JND

JNDs were not normally distributed (Shapiro–Wilk test, *p* < 0.05). The median JNDs derived from TOJs for active head movement paired with brief (44.35 ms, 25 % = 22.1, 75 % = 79.9), long square (66.5 ms, 25 % = 39.1, 75 % = 89.6) and long raised-cosine (95.0 ms, 25 % = 43.0, 75 % = 116.9) sounds are shown in Fig. 2b. Pairwise comparison tests confirmed that the significant effect of sound type on the JND (χ_(2)_^2^ = 10.5, *p* = 0.005) was driven by the long raised-cosine sound, which was significantly different from brief (*p* < 0.05) but not from long square (*p* > 0.05) sounds. The difference between long square and brief sounds was not significant (*p* > 0.5). These results indicate that, in general, participants were less precise when judging the timing of sound of a continuously changing intensity.

### Discussion

We originally speculated that the results of previous studies showing that vestibular stimulation must precede other sensory stimulation in order to be perceived as simultaneous (Barnett-Cowan and Harris 2009, 2011; Sanders et al. 2011) were attributable to the lacking equivalence in temporal envelope duration and shape of the brief pulses used and the longer vestibular signals. We more closely matched auditory stimuli to the vestibular signal and predicted that changing the stimuli to better match the vestibular signal would enhance participants’ ability to accurately perceive simultaneity and would therefore displace the PSS toward the point of true simultaneity. Instead of reducing this lead time, the time required for a head movement to precede an auditory stimulus actually increased by up to an additional 42 ms.

What can account for this additional lead time? Vos and Rasch (1981) posited a threshold model to understand the perceptual onset of musical tones. In order to perceive the onset of a tone, a certain perceptual threshold level, relative to the maximum amplitude, must be exceeded during the rise portion of the stimulus. An important factor influencing the timing of perceptual onsets is the rise time; for instance, if tones have simultaneous physical onsets but different rise times, the perceptual onsets will not occur simultaneously. A raised-cosine temporal envelope has a shallow slope, while a brief pulse has an extremely steep slope. According to the threshold model put forth by Vos and Rasch, the onset of the raised-cosine stimulus will be perceived as occurring later than the brief tone. As this can only lead to a pattern of results where the PSS would move toward true simultaneity, the threshold model cannot explain our results. Jaśkowski (1993), however, did find a curious but unexplained result where triangular stimuli that reached peak intensity earlier than mid-duration can be perceived as occurring before the onset of a square stimulus of equal duration. Although Jaśkowski did not provide an explanation for the effect, a mechanism responsible for it could also explain why a head movement would require additional lead time when preceding a raised-cosine stimulus to be synchronously perceived.

The influence of stimulus duration on processing time is more inconclusive in the literature. Jaśkowski (1991) showed that the onset of a shorter stimulus shifts toward the offset of its paired longer stimulus, causing a delay in processing of the shorter stimulus and thus a shift of the PSS (Jaśkowski 1991, 1992), but that this is largely diminished for discrepancies of more than 500 ms (Jaśkowski 1991). Efron (1970a, b, c), however, found no such effects of duration. Recently, Boenke et al (2009) attempted to resolve these inconsistencies in the literature. Contrary to the findings of Jaśkowski (1991, 1992) and consistent with Efron (1970a, b, c), their results established that duration does not change the PSS and therefore cannot account for discrepancies between auditory and visual processing. It should be noted, however, that when Boenke and colleagues assessed PSS values on an individual level, duration had an effect; however, the direction of this effect was not consistent across subjects, and thus, it is difficult to draw conclusions from this finding.

Given the inconsistency in the literature regarding the potential effects of rise time and duration on perceived temporal order, a second experiment was conducted by pairing the different sound stimuli with each other to assess whether possible significant differences between these stimuli could explain the results in experiment 1. In keeping with the results of Jaśkowski (1993), we predicted that a long square sound should be perceived as simultaneous with a long raised-cosine sound as the peak of the long raised-cosine sound occurs at the midpoint (i.e., not in the early portion) of the temporal envelope. We also predicted that a brief sound should be simultaneously perceived with a long square sound as would be expected by the observation of Jaśkowski (1991) that duration discrepancies greater than 500 ms should not affect the PSS.

## Experiment 2

### Procedure and data analysis

To confirm possible effects of stimulus temporal envelope duration and shape on the sound stimuli used in the present experiment, fourteen participants (four from the original study and 10 additional participants who also provided informed consent; four women, 22–31 years) completed a TOJ task comparing the auditory stimuli to each other using the same procedure and data analysis of the main experiment. Here, however, participants were instructed to indicate in which ear they perceived a sound first and responded with left and right button presses for left and right ears, respectively. Stimuli pairs were brief/long square, brief/long raised-cosine and long square/raised-cosine. Whether a given sound was presented to the left or right ear was counterbalanced across participants.

The percentage of responses in which sound presented to the right ear was selected as occurring first was plotted as a function of SOA, with negative SOAs signaling that the sound presented to the left ear occurred first. A two-parameter sigmoidal logistic curve (Eq. 2) was fitted to the data, and which ear a stimulus was first presented to from counterbalancing was accounted for. Statistical analysis included one-sample t-tests for the PSSs of each condition to confirm significant deviations from an SOA of 0. A one-way repeated-measures ANOVA was carried out to examine differences in the PSSs and JNDs due to temporal envelope duration and shape of the auditory stimuli.

### Results

**Fig. 3:**
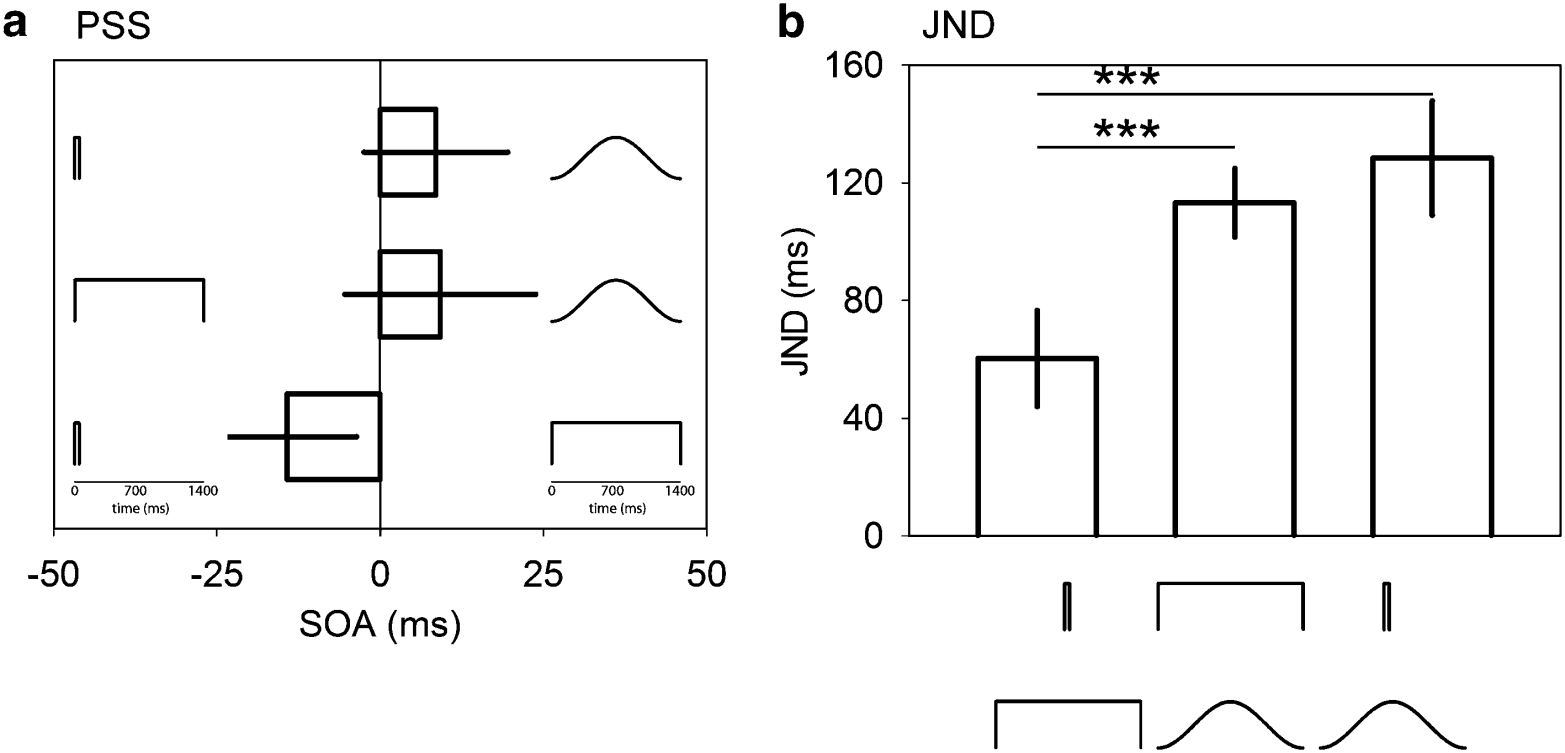
Experiment 2 results. **a** Average PSS plotted relative to SOA for each sound condition pairing. Note that the cartoons representing each sound condition have a separate *inset* timescale than that used for SOA. **b** Average JND data for each sound condition pairing. *Error bars* are ±1 SEM. ***p < 0.001

The average PSS results are shown in Fig. 3a. Here, the PSS did not significantly differ from an SOA of 0 (all *p* > 0.05) and no significant effect was found for sound condition (*F*_(2,28)_ = 2.19, *p* = 0.133), indicating that differences between the comparison stimuli cannot explain the results from experiment 1. A significant effect of sound condition was found, however, among the average JNDs (*F*_(2,28)_ = 19.15, *p* < 0.001). Here, pairwise comparison tests confirmed that participants were less precise when judging sound pairings containing the long raised-cosine stimulus, as the JNDs for long square–raised-cosine (128.4 ms, s.e. 19.4) and brief square–long raised-cosine (113.2 ms, s.e. 11.7) pairs were significantly higher (both *p* < 0.001) than the brief–long square pair (60.3 ms, s.e. 16.3). Note that this result is comparable to the JND results in experiment 1.

### Discussion

The PSS results in experiment 2 are in agreement with the work of Boenke et al. (2009) and Efron (1970a, b, c) and the observation of Jaśkowski (1991) regarding duration discrepancies greater than 500 ms, as we found that duration has no effect on the temporal processing of auditory stimuli. These results also confirm the result from experiment 1 where no significant difference in PSS was found between brief and long square sounds. The PSS results in experiment 2 also confirm the observation of Jaśkowski (1993) that a stimulus whose intensity peaks midway will not be perceived earlier than a square stimulus of similar duration. Also, our results are inconsistent with the work of Vos and Rasch (1981) whose threshold model would have predicted that the raised-cosine stimulus should be perceived as occurring later than a square stimulus of similar duration. We suggest that this may be due to the fact that our stimuli were relatively intense and rapidly rose above threshold.

In sum, matching the auditory and vestibular stimuli in experiment 1 resulted in an additional lead time of head movement prior to a sound. The results of experiment 2 indicate that differences within the comparison auditory stimulus do not account for this increased lead time. What then can account for the *additional* lead time required of head movement found in experiment 1?

#### Head movement variability

**Fig. 4:**
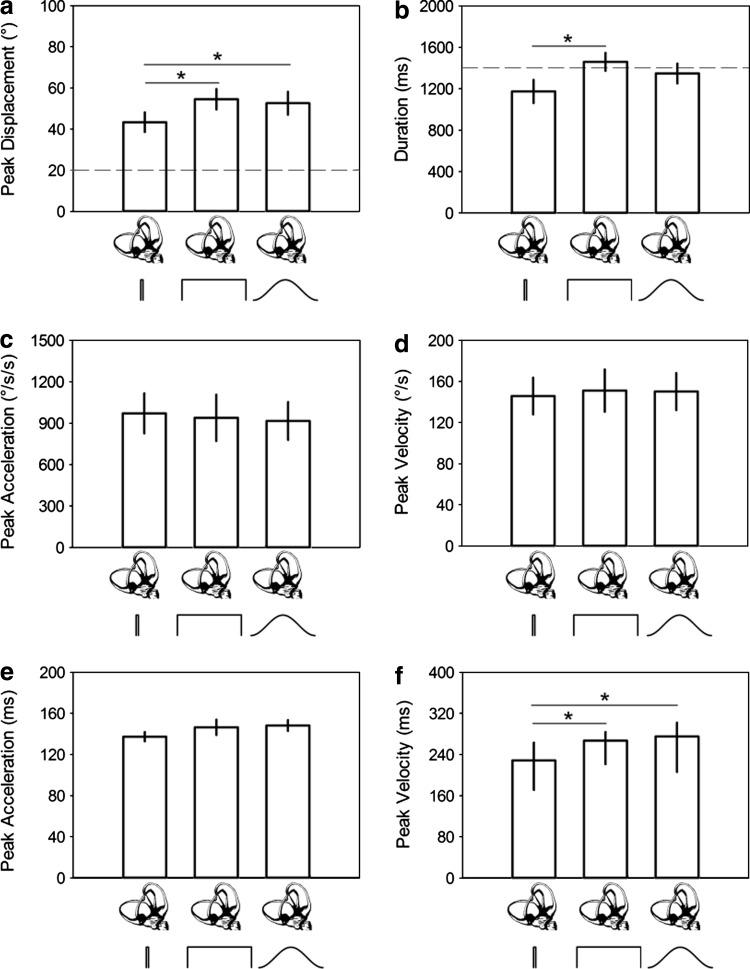
Head movement properties. Average peak displacement (**a**), average duration (**b**), median peak acceleration (**c**), average peak velocity (**d**), average time to reach peak acceleration (**e**) and median time to reach peak velocity (**f**) for each head movement–sound pairing. *Dashed lines* in **a** and **b** represent the target displacement and durations from training, respectively (see “General methods”). *Error bars* in **a**, **b**, **d** and **e** are ±1 SEM; 25th and 75th percentiles in **c** and **f**. *p < 0.05

To determine whether variability in the active head movements could explain this additional lead time, we analyzed the head movement properties in each condition from experiment 1 by first calculating the average head movement properties within each individual and then grouping them. On average, active head movement displacement was 50° (SD: 19.9), with a peak velocity of 149°/s (SD: 71.3) and peak acceleration of 941°/s/s (SD: 568.5). All head movement displacements were significantly greater (one-sample t-tests, all *p* < 0.001) than the 20° displacement to which participants were trained (Fig. 4a); however, average head movement duration was not significantly different from 1,400 ms for all sound conditions (all *p* > 0.05; Fig. 4b). Significant effects of sound type on the duration (*F*_(2,28)_ = 6.4, *p* = 0.005; Fig. 4a) and displacement (*F*_(2,28)_ = 7.5, *p* = 0.002; Fig. 4b) of head movement were found. Pairwise comparison tests confirmed that this was driven primarily by the brief square sound; head movement in this condition was significantly shorter in duration compared to long square sound (*p* < 0.05) and shorter in displacement compared to the other sound conditions (all *p* < 0.05). However, no significant effect of sound type was found on head movement peak acceleration (χ_(2)_^2^ = 2.8; *p* = 0.247; Fig. 4c) or velocity (*F*_(2,28)_ = 0.2, *p* = 0.856; Fig. 4d)—which is more relevant for information pertaining to head movement onset.

On average, the time to reach peak acceleration was 143.9 ms (s.e. 3.8), but no differences were found between conditions (*F*_(2,28)_ = 1.7, *p* = 0.197; Fig. 4e). There was, however, a significant effect of condition on the time to reach peak velocity (χ_(2)_^2^ = 20.9, *p* < 0.001; Fig. 4f), such that median peak velocity was reached in 228.5 ms (25 % = 170.9, 75 % = 264.9) for brief square sound, which was significantly faster than that for long square 266.9 ms (25 % = 219.6, 75 % = 285.1) and raised-cosine 275.0 ms (25 % = 205.9, 75 % = 302.1) sounds (both *p* < 0.05).

**Fig. 5:**
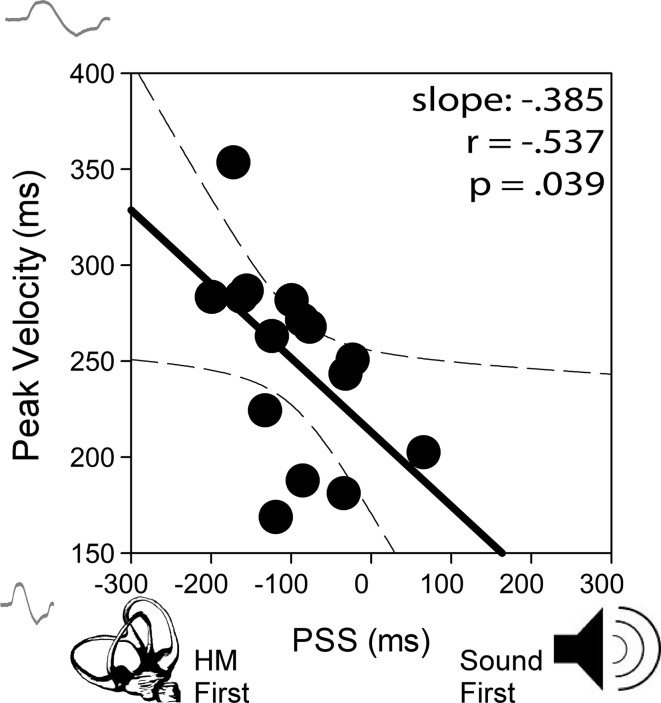
Average time to reach peak velocity as a function of PSS. A significant negative correlation here indicates that delays in reaching peak velocity largely account for the additional head movement lead time (negative shift of the PSS) that was found among sound conditions. Note that most data points are in the negative direction relative to 0. *Dashed lines* represent 95 % confidence intervals

Correlation analysis comparing head movement properties with the PSS revealed a significant negative correlation between the time to reach peak velocity and the PSS (*r*_(*15*)_ = −0.537, *p* = 0.039; Fig. 5). Note that data across the sound conditions were pooled within each participant and then entered into the Pearson’s product–moment correlation as suggested by Bland and Altman (1994) in order to account for repeated data. This result, paired with results from experiment 2, showing no PSS differences between the sound stimuli, suggests that no *additional* lead time is required of head movement when paired with a sound of similar temporal envelope duration and shape.

## General discussion

Matching the properties of auditory and vestibular stimuli does not account for the 73 ms lead time required of a head movement to be perceived as simultaneous with a brief sound. If anything, the attempt to equate stimulus properties led to an *additional* lead time of up to 42 ms. However, this additional lead time was found to be attributable to differences across conditions in the time to reach peak head movement velocity, where a slower rise in velocity denotes slower detection of head movement onset. While participants were trained to make head movements prior to each condition, it is possible that participants were inclined to mimic the sounds in order to best match the stimulus pair, particularly as the block design used would have allowed participants to anticipate the sound type within a given block. This could explain a quicker time to reach peak velocity when moving their head when paired with brief sounds and a slower time when paired with long sounds.

Additional task demands of having to execute head movements as well as having to monitor head movement and sound onset may have contributed to the poor replication of head movement velocity from training to experimental trials. It is also possible that replicating head movement speed in the absence of visual feedback, which was present in training but absent during experimental trials, led to faster head movements during experimental trials. Head movements during the training and experimental phases can be considered as tracking and ballistic, respectively, which are known to recruit different muscle group combinations (Peterson 2004). However, as peak velocity did not significantly differ across the sound condition pairings and head movements during experimental trials were all ballistic, we do not think that differential activation of neck muscles can explain perceptual differences in head movement onset. Finally, participants may have made faster head movements in an effort to produce a stronger signal with which to compare auditory stimuli. We propose that real-time feedback of head tracking during training, and post hoc removal of unsuitable traces drawn from a larger sample of experimental trials should be employed to avoid this issue in future experiments (see Leung et al. 2008).

Why is it, however, that the perceived timing of head movement changes with the rise time of head movement velocity? One possible explanation is that change in the perceived timing of natural head movements is determined by the dynamics of peripheral mechanisms. In a previous paper (Barnett-Cowan and Harris 2011), it was found that change in the PSS of active head movements paired with a touch, light or sound was very similar to the rate of suppression of vestibular afferent signals as reported by Roy and Cullen (2004) from electrophysiological recordings in monkeys. With respect to the present paper, change in the perceived timing of head movement with the rise time of head movement velocity may be attributable to increase in neural firing gain and a phase lead of vestibular afferent neurons that have been observed for increasingly rapid rotations of the head (Fernandez and Goldberg 1971). Indeed, it was Fernandez and Goldberg (1971) who speculated that such a phase lead could be used to potentially compensate for neural delays upstream. Given the observational similarities between the results in the present paper and those from electrophysiological recordings of monkey vestibular afferent neurons, the extent to which the dynamics of the perceived timing of natural head movements are determined by peripheral mechanisms deserves future direct investigation.

An alternative explanation could be that head movement onset is ill-defined. It is entirely possible that participants did not estimate their head movements relative to their onset but to some other cue such as peak acceleration. This concern was addressed in a previous paper assessing active versus passive head movements paired with touch, light or sound (Barnett-Cowan and Harris 2011). Here, it was found that the time that an active or passive head movement takes to reach peak acceleration is the same (around 80 ms) despite an active head movement requiring around 80 ms to precede other stimuli while a passive head movement requires around 45 ms lead time to be perceived synchronously with other stimuli. As no differences in the time to reach peak acceleration were found between conditions in the present paper, it is unlikely that participants used another cue such as peak acceleration to make their judgments. Still, as the time to reach peak velocity was found to be a physical correlate of perceived head movement onset, the definition of head movement onset remains questionable. We suggest that future experiments should be conducted where participants are asked to judge the perceived timing of events relative to different time points of a head movement. It is important to note that all head movements rose rapidly above the threshold to detect rotation of the head (~1°/s: Soyka et al. 2012). As such, the differences observed across sound conditions in the time it took to reach peak velocity can only explain change in the additional lead time required for the head to precede sound, and they cannot explain the constant amount of lead time of approximately 73 ms required of the head to precede a sound in order to be synchronously perceived. This unexpected delay in the perceived timing of vestibular stimulation thus remains surprising, considering the speed with which the vestibular system detects and responds to self-motion.

The persistence of the delay in the perceived timing of vestibular stimulation found in the present study provides compelling evidence that central processing of vestibular signals most likely accounts for the delayed awareness of vestibular input. The brain has been shown to be able to compensate for differential physical and neural delays through central processing (Engel and Dougherty 1971; Sugita and Suzuki 2003; Kopinska and Harris 2004), yet compensation for vestibular stimulation is only partial (Barnett-Cowan and Harris 2009). The inability of the brain to fully compensate for these delays may reflect the fact that vestibular stimulation is most often associated with sensory events that occur following head movement. In addition, the vestibular system rarely acts alone; its signals at the level of the cortex are highly distributed (de Waele et al. 2001; Bense et al. 2001), and this may prevent access to direct conscious awareness of vestibular stimulation (Angelaki and Cullen 2008). Here, it is interesting to note that additional proprioceptive information from the neck muscles, which are very much available for the rapid head movements used in this and in a previous study (Barnett-Cowan and Harris 2011), does not provide enough additional information to overcome this perceptual delay. Further, because perception and motor planning require information about the position of the head and not its velocity—which is what the vestibular afferent signal is proportional to (Fernandez and Goldberg 1971)—an integration is required whose sampling time could account for perceptual latencies reported in the literature and the present paper. Finally, the brain may prioritize physiological response to vestibular stimulation over perceptual awareness of stimulation onset in order to maintain perceptual and postural stability. This has been shown in control tasks such as when having to control a helicopter, where vestibular information provides information for stability control faster than visual stimuli (Berger et al. 2007; Berger and Bülthoff 2009). It is also arguably more important to brace oneself during a fall then being able to recollect when the fall began. Of note, it has recently been reported that those who are fall-prone are poor at judging the relative timing of events (Setti et al. 2011). Additional investigations are therefore required to determine the extent to which these possible explanations can account for delays in the perceived timing of vestibular stimulation.
